# Supplementary material for: Unveiling the Bioactive Potential of the Invasive Jellyfish Phyllorhiza punctata Through Integrative Transcriptomic and Proteomic Analyses
Source: Biomolecules. 2025 Aug 4;15(8):1121. doi: 10.3390/biom15081121 (PMC12383608; doi:10.3390/biom15081121)
Supplement: Supplementary file 1 [file biomolecules-15-01121-s001.zip › Suplementary Material/FileS3/PEPFOLD4/XP_065055434_10500/clusters.html]

 PEP-FOLD clusters


Download archive of all models  here   

|  |  |  |
| --- | --- | --- |
| # | file | sOPEP |
| # cluster 1 - Size: 2 |  |  |
|  | XP\_065055434\_10500-model1 | -2.21579 |
|  | XP\_065055434\_10500-model1.1 | -1.83727 |
| # cluster 2 - Size: 7 |  |  |
|  | XP\_065055434\_10500-model2 | -2.19516 |
|  | XP\_065055434\_10500-model2.1 | -2.1683 |
|  | XP\_065055434\_10500-model2.2 | -2.05008 |
|  | XP\_065055434\_10500-model2.3 | -1.99348 |
|  | XP\_065055434\_10500-model2.4 | -1.95704 |
|  | XP\_065055434\_10500-model2.5 | -1.95269 |
|  | XP\_065055434\_10500-model2.6 | -1.9267 |
| # cluster 3 - Size: 2 |  |  |
|  | XP\_065055434\_10500-model3 | -2.16662 |
|  | XP\_065055434\_10500-model3.1 | -1.86907 |
| # cluster 4 - Size: 3 |  |  |
|  | XP\_065055434\_10500-model4 | -2.14743 |
|  | XP\_065055434\_10500-model4.1 | -1.98485 |
|  | XP\_065055434\_10500-model4.2 | -1.83086 |
| # cluster 5 - Size: 2 |  |  |
|  | XP\_065055434\_10500-model5 | -2.10808 |
|  | XP\_065055434\_10500-model5.1 | -1.93249 |
| # cluster 6 - Size: 7 |  |  |
|  | XP\_065055434\_10500-model6 | -2.09267 |
|  | XP\_065055434\_10500-model6.1 | -2.06547 |
|  | XP\_065055434\_10500-model6.2 | -2.0626 |
|  | XP\_065055434\_10500-model6.3 | -2.05162 |
|  | XP\_065055434\_10500-model6.4 | -2.03309 |
|  | XP\_065055434\_10500-model6.5 | -2.00141 |
|  | XP\_065055434\_10500-model6.6 | -1.978 |
| # cluster 7 - Size: 4 |  |  |
|  | XP\_065055434\_10500-model7 | -2.08106 |
|  | XP\_065055434\_10500-model7.1 | -2.05557 |
|  | XP\_065055434\_10500-model7.2 | -2.02988 |
|  | XP\_065055434\_10500-model7.3 | -1.98992 |
| # cluster 8 - Size: 1 |  |  |
|  | XP\_065055434\_10500-model8 | -2.0489 |
| # cluster 9 - Size: 1 |  |  |
|  | XP\_065055434\_10500-model9 | -2.04455 |
| # cluster 10 - Size: 3 |  |  |
|  | XP\_065055434\_10500-model10 | -2.03906 |
|  | XP\_065055434\_10500-model10.1 | -1.92475 |
|  | XP\_065055434\_10500-model10.2 | -1.89914 |
| # cluster 11 - Size: 9 |  |  |
|  | XP\_065055434\_10500-model11 | -2.03758 |
|  | XP\_065055434\_10500-model11.1 | -2.01115 |
|  | XP\_065055434\_10500-model11.2 | -1.98449 |
|  | XP\_065055434\_10500-model11.3 | -1.94686 |
|  | XP\_065055434\_10500-model11.4 | -1.9054 |
|  | XP\_065055434\_10500-model11.5 | -1.88854 |
|  | XP\_065055434\_10500-model11.6 | -1.86892 |
|  | XP\_065055434\_10500-model11.7 | -1.8304 |
|  | XP\_065055434\_10500-model11.8 | -1.81754 |
| # cluster 12 - Size: 3 |  |  |
|  | XP\_065055434\_10500-model12 | -2.03102 |
|  | XP\_065055434\_10500-model12.1 | -1.98819 |
|  | XP\_065055434\_10500-model12.2 | -1.95672 |
| # cluster 13 - Size: 4 |  |  |
|  | XP\_065055434\_10500-model13 | -2.02001 |
|  | XP\_065055434\_10500-model13.1 | -2.00321 |
|  | XP\_065055434\_10500-model13.2 | -1.93946 |
|  | XP\_065055434\_10500-model13.3 | -1.92342 |
| # cluster 14 - Size: 1 |  |  |
|  | XP\_065055434\_10500-model14 | -1.95275 |
| # cluster 15 - Size: 15 |  |  |
|  | XP\_065055434\_10500-model15 | -1.9462 |
|  | XP\_065055434\_10500-model15.1 | -1.91888 |
|  | XP\_065055434\_10500-model15.2 | -1.90534 |
|  | XP\_065055434\_10500-model15.3 | -1.89778 |
|  | XP\_065055434\_10500-model15.4 | -1.88602 |
|  | XP\_065055434\_10500-model15.5 | -1.88571 |
|  | XP\_065055434\_10500-model15.6 | -1.8828 |
|  | XP\_065055434\_10500-model15.7 | -1.86969 |
|  | XP\_065055434\_10500-model15.8 | -1.86949 |
|  | XP\_065055434\_10500-model15.9 | -1.86857 |
|  | XP\_065055434\_10500-model15.10 | -1.8618 |
|  | XP\_065055434\_10500-model15.11 | -1.85781 |
|  | XP\_065055434\_10500-model15.12 | -1.85341 |
|  | XP\_065055434\_10500-model15.13 | -1.84428 |
|  | XP\_065055434\_10500-model15.14 | -1.83482 |
| # cluster 16 - Size: 1 |  |  |
|  | XP\_065055434\_10500-model16 | -1.91536 |
| # cluster 17 - Size: 3 |  |  |
|  | XP\_065055434\_10500-model17 | -1.91299 |
|  | XP\_065055434\_10500-model17.1 | -1.88675 |
|  | XP\_065055434\_10500-model17.2 | -1.86142 |
| # cluster 18 - Size: 5 |  |  |
|  | XP\_065055434\_10500-model18 | -1.9129 |
|  | XP\_065055434\_10500-model18.1 | -1.87833 |
|  | XP\_065055434\_10500-model18.2 | -1.87471 |
|  | XP\_065055434\_10500-model18.3 | -1.84654 |
|  | XP\_065055434\_10500-model18.4 | -1.82794 |
| # cluster 19 - Size: 7 |  |  |
|  | XP\_065055434\_10500-model19 | -1.90366 |
|  | XP\_065055434\_10500-model19.1 | -1.8843 |
|  | XP\_065055434\_10500-model19.2 | -1.86574 |
|  | XP\_065055434\_10500-model19.3 | -1.86319 |
|  | XP\_065055434\_10500-model19.4 | -1.85667 |
|  | XP\_065055434\_10500-model19.5 | -1.8241 |
|  | XP\_065055434\_10500-model19.6 | -1.78549 |
| # cluster 20 - Size: 1 |  |  |
|  | XP\_065055434\_10500-model20 | -1.90018 |
| # cluster 21 - Size: 2 |  |  |
|  | XP\_065055434\_10500-model21 | -1.87958 |
|  | XP\_065055434\_10500-model21.1 | -1.87796 |
| # cluster 22 - Size: 2 |  |  |
|  | XP\_065055434\_10500-model22 | -1.8736 |
|  | XP\_065055434\_10500-model22.1 | -1.82466 |
| # cluster 23 - Size: 4 |  |  |
|  | XP\_065055434\_10500-model23 | -1.84898 |
|  | XP\_065055434\_10500-model23.1 | -1.84212 |
|  | XP\_065055434\_10500-model23.2 | -1.8263 |
|  | XP\_065055434\_10500-model23.3 | -1.82242 |
| # cluster 24 - Size: 2 |  |  |
|  | XP\_065055434\_10500-model24 | -1.84106 |
|  | XP\_065055434\_10500-model24.1 | -1.82112 |
| # cluster 25 - Size: 1 |  |  |
|  | XP\_065055434\_10500-model25 | -1.83626 |
| # cluster 26 - Size: 7 |  |  |
|  | XP\_065055434\_10500-model26 | -1.83592 |
|  | XP\_065055434\_10500-model26.1 | -1.82174 |
|  | XP\_065055434\_10500-model26.2 | -1.81677 |
|  | XP\_065055434\_10500-model26.3 | -1.81509 |
|  | XP\_065055434\_10500-model26.4 | -1.81338 |
|  | XP\_065055434\_10500-model26.5 | -1.8103 |
|  | XP\_065055434\_10500-model26.6 | -1.7993 |
| # cluster 27 - Size: 1 |  |  |
|  | XP\_065055434\_10500-model27 | -1.82665 |
|  |  |  |
